# Synergetic effect of the surface ligand and SiO_2_ driven photoluminescence stabilization of the CH_3_NH_3_PbBr_3_ perovskite magic-sized clusters

**DOI:** 10.1038/s41598-021-01560-4

**Published:** 2021-11-15

**Authors:** Fitri Aulia Permatasari, Hilma Eka Masitoh, Ea Cahya Septia Mahen, Bebeh Wahid Nuryadin, Akfiny Hasdi Aimon, Yana Maolana Syah, Ferry Iskandar

**Affiliations:** 1 Department of Physics, Faculty of Mathematics and Natural Sciences, Institut Teknologi Bandung, Jalan Ganesha 10, Bandung, West Java 40132 Indonesia; 2 Department of Physics Education, Faculty of Tarbiyah and Education, UIN Sunan Gunung Djati Bandung, Jl. A. H. Nasution 105, Bandung, 40614 Indonesia; 3 Department of Physics, Faculty of Science and Technology, UIN Sunan Gunung Djati Bandung, Jl. A. H. Nasution 105, Bandung, 40614 Indonesia; 4 Department of Chemistry, Faculty of Mathematics and Natural Sciences, Institut Teknologi Bandung, Jalan Ganesha 10, Bandung, 40132 Indonesia; 5 Research Center for Nanoscience and Nanotechnology, Institut Teknologi Bandung, Jalan Ganesha 10, Bandung, 40132 Indonesia

**Keywords:** Materials science, Quantum dots, Synthesis and processing

## Abstract

Zero-dimensional Perovskite Magic-size Clusters play crucial roles in understanding and controlling nucleation and growth of semiconductor nanoparticles. However, their metastability behavior is a critical hindrance for reliable characterizations. Here, we report the first demonstration of using an excess amount of surface ligand and SiO_2_ as novel passivation for synthesizing the magic-sized clusters (MSCs) by the Ligand-assisted reprecipitation method. A synergetic effect between an excessed surface ligand and SiO_2_ inhibits the protonation and deprotonation reaction between amine-based and acid-based ligand, leading to enhanced PL stability. The obtained CH_3_NH_3_PbBr_3_ PMSCs/SiO_2_ retain 70% of its initial emission intensity in ambient conditions for 20 days. This passivation approach opens an entirely new avenue for the reliable characterizations of CH_3_NH_3_PbBr_3_ PMSCs, which will significantly broaden their application for understanding and controlling nucleation and growth of semiconductor nanoparticles.

## Introduction

The finding of so-called Magic-size clusters (MSCs) is now one of the most intriguing nanoparticles research outcomes. An accelerated development could be achieved by fully understanding the MSCs, owing to their attributed roles as intermediates in nanoparticles' formation. MSCs usually be associated with the ultrasmall nanoparticle with narrow distribution sized and a strong quantum confinement effect^1^. Compared with the conventional nanoparticles, *e.g.*, Quantum Dots (QDs), MSCs possessed unique properties such as smaller size than QDs with narrow distribution, narrow photoluminescence (PL) and narrow absorption peaks owing to their molecule-like behaviour^2–4^. MSCs perform the optical properties that notably red-shift in discrete steps as the reaction progresses before the particle growth turns into the conventional QDs. These properties have corresponded to their structure that assumes be a closed shell configuration with well-defined stoichiometry, shape, and size, as well as to occupy deep, local minima in the potential landscape.

An elusive structure and growth mechanism of the PQDs originated from MSCs has become a major obstacle for further developing the PQDs^5–7^. Thus, Perovskite MSCs (PMSCs) serve as an appropriate model system for elucidating the PQDs formation mechanism, as well as their metastability and surface-related issue^8^. However, their metastability structure hinder the elucidation process that mainly relies on the reliable characterization of MSCs. The metastability behaviour of PMSCs owing to the abundant charging defects in perovskite surface, including positive-charge defect (i.e., halide ion vacancy) and negative-charge defects (i.e., cation vacancy and PbX_4_^2−^ anti-site defects)^8^. The surface passivation MSCs by various capping ligands, including organic acid and amines have been reported^9,10^. It was demonstrated that the PMSCs and PQD structure could be tunning by adjusting the ligand concentration ratio between acid and amines-based ligands implying the crucial role of the ligands to the MSCs formation^9,10^. Unfortunately, the dynamic nature of the interaction between PMSCs surface and capping ligands permits the ligand detachment from the PMSCs surface under various conditions^11^. A sample preparation^9^, including vacuum conditioning^10,12^, drying process^13^, or even simple purification steps that alter the samples' ligand concentrations^14^, may disrupt the initial PMCS structure concerning the reliability characterization. The ligand's highly dynamic binding state that strongly correlated to their concentration might result in various complex problems in those sample preparation. However, the conclusive mechanism of this interaction is a long, hard struggle to be solved owing to their complex interactions and a serious complication in characterization. Furthermore, it remains unclear whether the surface ligands are passivating the existing trap states or developing new ones inducing a severely PL quenching. Therefore, another strategic approach for synthesized the PMSCs with good long-term stability for reliable characterization should be considered.

Silica (SiO_2_) is a versatile material usually treated as a prospective candidate to resolve the long-standing bottleneck stability issue in nanostructure materials^11,15,16^. We note that the silica encapsulation has been well documented over the last decade using in-situ and ex-situ synthetic routes to enhancing the stability of the PQDs^17–21^. However, all these methods still suffer their drawbacks that hinder the practical applications of PQDs. For example, the traditional silica coating process produced only thick shells that gather a large volume of QDs, incurring difficulties in coupling them with conventional optoelectronic platforms. Various integration processes of SiO_2_ into PQDs structure generate weak interaction between SiO_2_ and PQDs surfaces preserve a new surface state, reducing PL emission^22–24^. In contrast, Nakamura et al. reported that the Si capping layer on the QDs structure could serve a radiative defect state that is a plausible mechanism for enhancing the QDs’ PL intensity^25^. Role of the Si to the PL properties of QDs was strongly attributed to the QD structure, including geometry and strain stress^25^. Therefore, it still highly desirable to endow SiO_2_ incorporated perovskite nanostructure especially in Magic-sized cluster phase with long-term stability and elucidate their interaction. To the best of our knowledge, there has been no study reported on the SiO_2_ interaction with PMSCs with enhanced PL stability.

Herein, taking advantage of an excessive amount of organic and amine-based ligands synergetic with the SiO_2_ incorporation on the PMSCs surface, we developed a facile approach to produce the CH_3_NH_3_PbBr_3_ PMSCs with good stability. We evaluated the effect of the SiO_2_ incorporation on the optical properties of the PMSCs. Intriguingly, a synergetic effect of surface ligand and SiO_2_ incorporation on PMSCs enhanced the PL stability under ambient temperature for 20 days without any significant change in size and optical properties of PMSCs. This study suggests that the obtained CH_3_NH_3_PbBr_3_ PMSCs/SiO_2_ with good stability is preferable as a good model, representing actual morphological and structural PMSCs to study both of PMSCs and PQDs structural and formation mechanism through a reliable characterization.

## Results

### Optical properties of CH_3_NH_3_PbBr_3_ PMSCs and CH_3_NH_3_PbBr_3_ PMSCs/SiO_2_

The perovskite samples were prepared using a ligand assisted reprecipitation (LARP) method as described in previous reports^10,26^. Excessive surface ligands comprising oleylamine and oleic acid are used as capping ligands to produce the ultrasmall size of interest^9,10^. The PL emission of the samples precipitated in room temperature exhibits a blue shift emission from 550 nm up to 450 nm in varying the ligand concentration ratio (Fig. S1). A higher concentration of Oleylamine ligand in a fixed concentration of Oleic acid, inducing a blue shift emission (Fig. S2) that was also observed and investigated comprehensively in other report^10^. It was probably due to the smaller size of the perovskite that strongly corresponds to the coordination these two ligands^10^. However, the PL intensity of the samples was decreased as increasing the Oleylamine concentration (Fig. S3). However, that a high ligand concentration could hinder the practical application owing to the long-term problem during drying process. Then, the 1:0.1 ligand concentration was selected to be further investigation owing to its high PL intensity.

**Figure 1:**
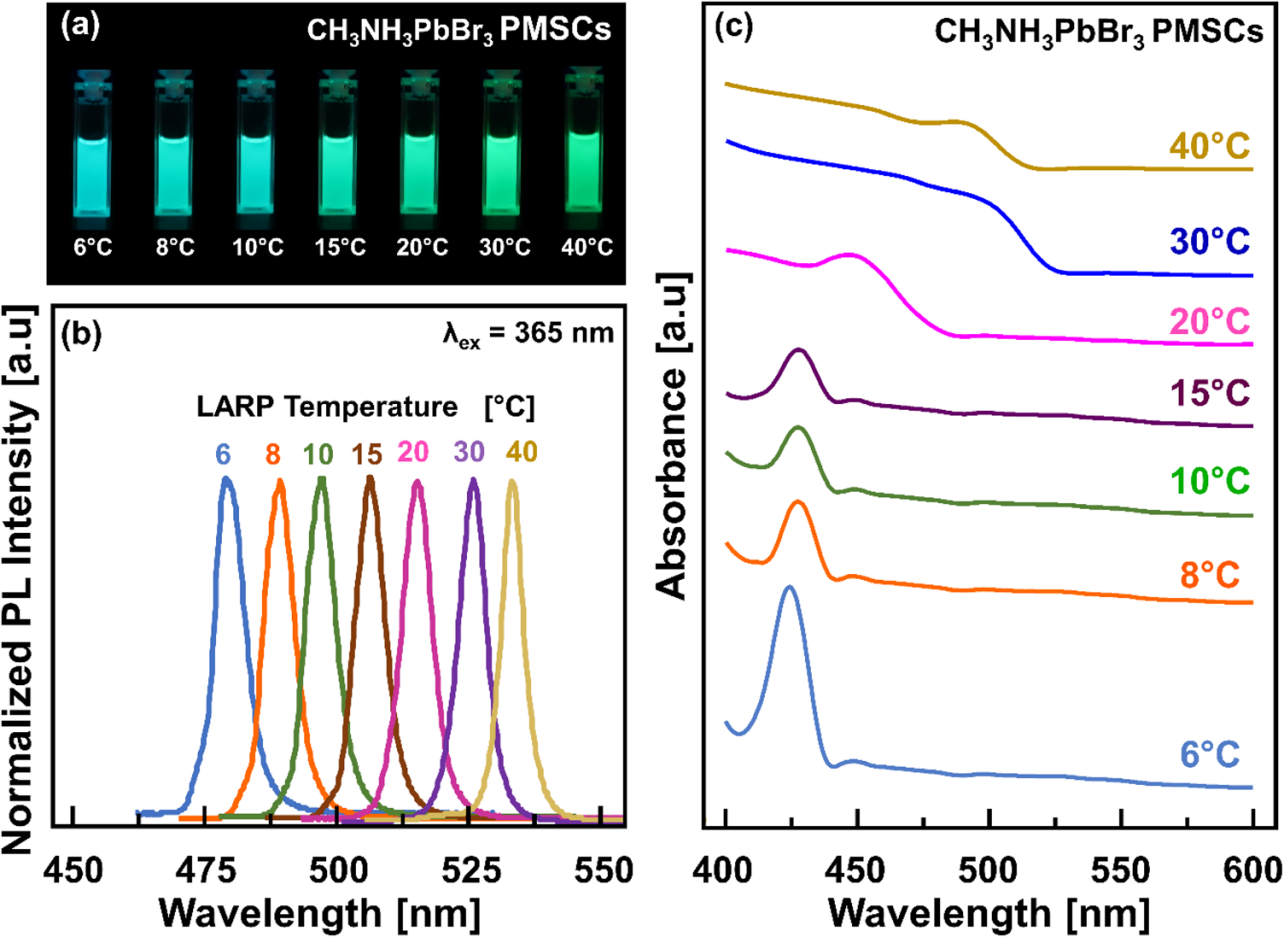
Optical properties of the as-synthesized CH_3_NH_3_PbBr_3_ PMSCs in varying precipitation temperature. (**a**) Digital photograph of the luminescence of samples under 365 nm UV irradiation. (**b**) PL spectra of all samples under 365 nm UV excitation. (**c**). Optical absorption spectra of the samples.

In varying the precipitation temperature, the solution colour turned into a bright yellow and subsequently exhibited a luminous blue to green under 365 nm UV lamp irradiation, as shown in Fig. 1a. Their photoluminescence (PL) spectra that are shown in Fig. 1b presented that all samples exhibited a single narrow emission peak centred at 480–530 nm wavelength (blue-green light). A narrow emission peak was redshifted in increasing the precipitation temperature that was also reported in other reports^26^. Figure 1c presents the UV–Vis absorption spectra of all prepared samples 10-folds diluted in toluene. In the case of precipitation temperature of 6 °C, only one excitonic absorption peak at 423 nm was observed and remained unchanged even after the temperature was further increased to 15 °C. As increasing the temperature up to 40 °C, broader absorption spectra with a band edge in the range of 480–520 nm were observed. A redshifted near band of its absorption spectra as increasing the precipitation temperature indicating the larger size of the perovskite^27^. The full width at half-maximum (FWHM) of its absorption band is around 12 nm, and the PL band is 25 nm, indicating a narrow distribution size. It suggested that the broader absorption spectra corresponded to the PQDs as reported in other reports^26,28,29^, while the single sharp excitonic peaks at 423 nm are attributed to the PMSCs as well observed in other reports literatures^9,10,30^.

**Figure 2:**
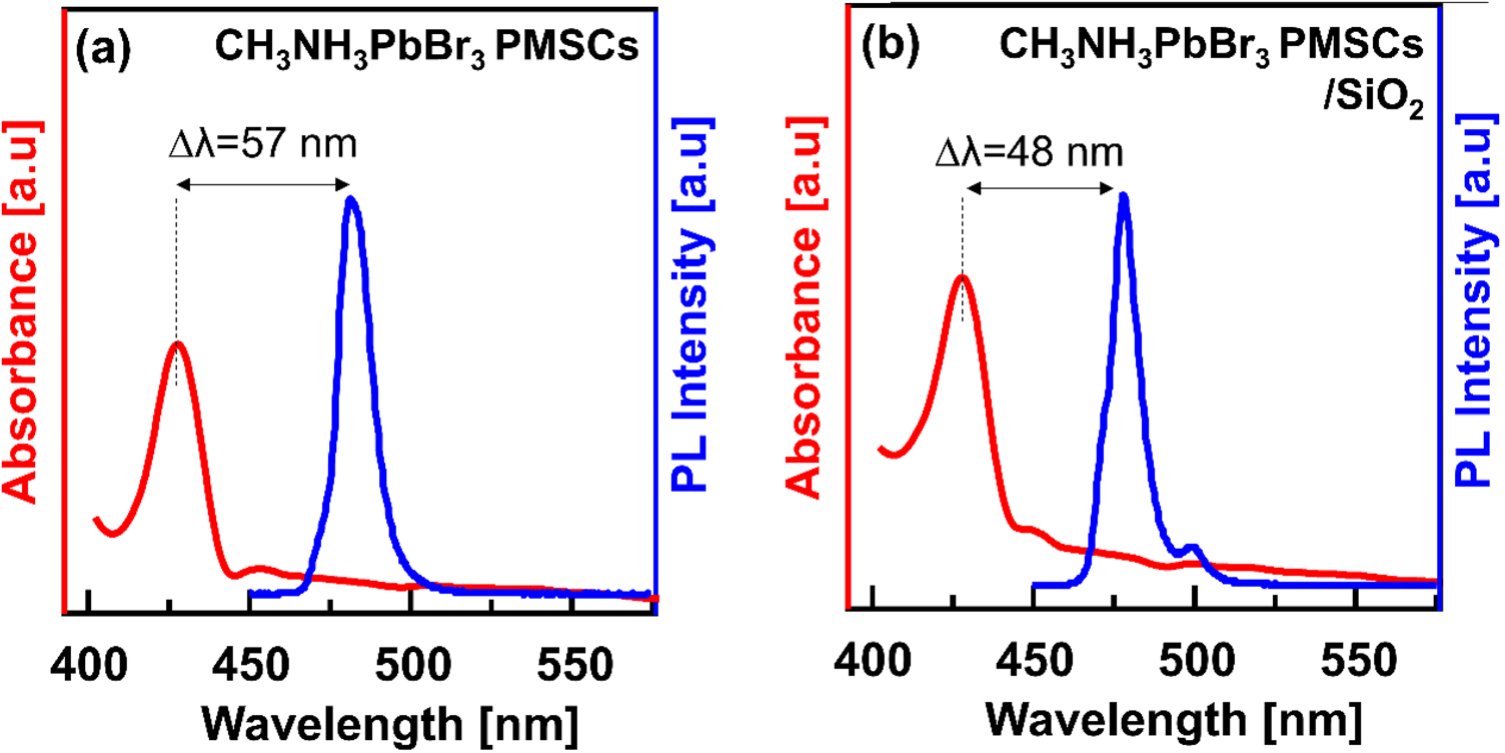
UV–Vis absorption and PL emission of the as-synthesized samples that synthesized at 6 °C. (**a**) CH_3_NH_3_PbBr_3_ PMSCs. (**b**) CH_3_NH_3_PbBr_3_ PMSCs/SiO_2_.

Figure 2a presents the UV–Vis absorption and PL spectrum of the CH_3_NH_3_PbBr_3_ PMSCs that synthesized at 6 °C. A relatively narrow emission spectrum (FWHM ~ 25 nm) indicating a very high colour purity that a preferable property for the display application^21^. A Stokes shifted of 57 nm might be attributed to the direct recombination process^26^. Despite a bright and tunable emission, the as-synthesized PMSCs are in a metastable state owing to their molecular-like behaviour. Then, a SiO_2_ was used as a binder during the precipitation process of the PMSCs. The UV–Vis absorption and PL spectra of the CH_3_NH_3_PbBr_3_ PMSCs/SiO_2_ are shown in Fig. 2b. Intriguingly, a reduced Stokes shift (from 57 to 48 nm) of the PL emission relative to the excitonic absorption peak was observed. On the molecular-like particle, the stokes shift is linearly dependent on the Huang-Rhys factor (S), corresponding to the correlation between electron-vibrational coupling and chain length^31^. Empirically, *S* = *a exp (-n*^*2*^*/b)* where *a* and *b* are arbitrary constants and *n* is the number of atoms in the molecular system^31,32^. Then, the reduced stokes shifted of the CH_3_NH_3_PbBr_3_ PMSCs/SiO_2_ could possibly indicate the more rigid molecular structure induced by a larger molecular system or increased chain length on the surface of PMSCs^33,34^. It was reasonable with the presence of the SiO_2_ during the precipitation process of the CH_3_NH_3_PbBr_3_ PMSCs which indicate the incorporation of the SiO_2_ to the PMSCs structure. The details of the incorporation of SiO_2_ to the PMSCs structure will be further discussed. Intriguingly, a new shoulder PL emission appeared at 500 nm, attributing to the trap state of the CH_3_NH_3_PbBr_3_ PMSCs composite or the smaller size PQDs formed^10^. However, a shoulder PL spectrum has not been observed for the CH_3_NH_3_PbBr_3_ PMSCs without SiO_2_ even after several days (Fig. S4). These results clearly point to the contribution of the SiO_2_ nanoparticles to the shoulder emission peak of the CH_3_NH_3_PbBr_3_ PMSCs.

### Morphology and energy level of CH_3_NH_3_PbBr_3_ PMSCs and CH_3_NH_3_PbBr_3_ PMSCs/SiO_2_

**Figure 3:**
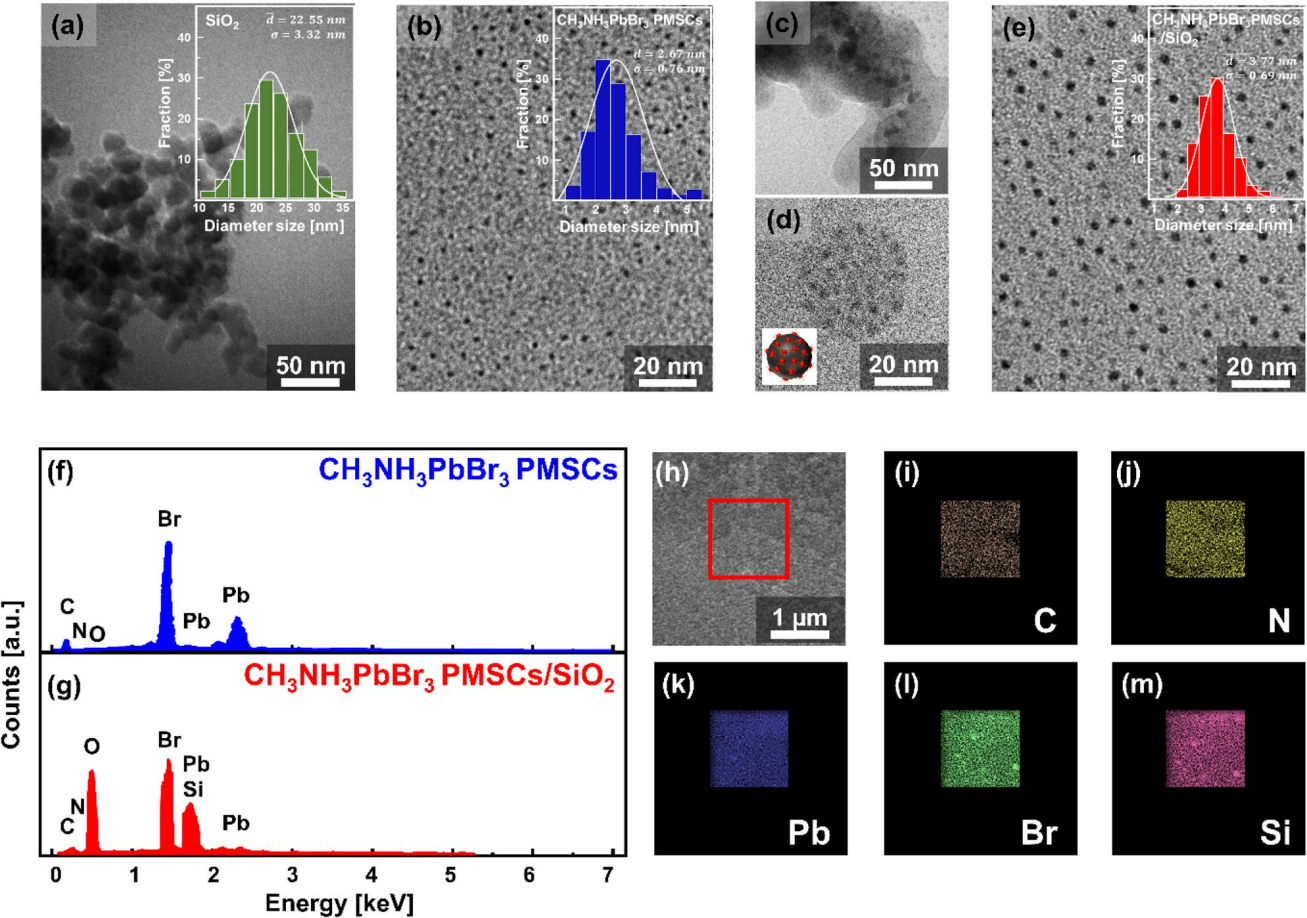
TEM images and its corresponded size distribution of the (**a**) SiO_2_, (**b**) CH_3_NH_3_PbBr_3_ PMSCs, (**c**–**e**) CH_3_NH_3_PbBr_3_ PMSCs/SiO_2_. EDS spectra of the (**f**) CH_3_NH_3_PbBr_3_ PMSCs, (**g**) the CH_3_NH_3_PbBr_3_ PMSCs/SiO_2_. (**h**–**m**) EDS mapping analysis of the CH_3_NH_3_PbBr_3_ PMSCs/SiO_2_ showing the elemental distribution of carbon, nitrogen, lead, bromine, and silicon, respectively.

TEM measurement of the representative samples was conducted to evaluate the structural and morphological samples. The SiO_2_ that used (Fig. 3a) shows spherical in agglomerated condition in diameter size of 22.55 ± 3.32 nm. In contrast, the CH_3_NH_3_PbBr_3_ PMSCs sample (Fig. 3b) shows spherical dots in a uniform distribution in size of from 2.67 ± 0.67 nm. In the presence of the SiO_2_ during the precipitation process, the CH_3_NH_3_PbBr_3_ PMSCs was well composed with the SiO_2_ (Fig. 3c). Interestingly, the PMSCs were well-distributed over the SiO_2_ surfaces indicating the good synergetic effect of ligands with the SiO_2_ surfaces (Fig. 3d). Furthermore, in the presence of the SiO_2_, the PMSCs morphology are remained unchanged in spherical shapes with average diameter size of 3.77 ± 0.69 nm (Fig. 3e). The estimated diameter of the CH_3_NH_3_PbBr_3_ PMSCs was calculated based on the Brus equation on the order of 2–4 nm^9^. While the CH_3_NH_3_PbBr_3_ PMSCs size reported on the other previous reports commonly shows a slightly larger diameter size (5.0 ± 0.9 nm) than the estimated value that was calculated from Bruss model (around 2–4 nm) due to the aggregation induced by the electron microscopy preparations that could not be avoided^10^. Despite the shortcomings of the TEM measurement tools with regards to providing the precise structural and information of the Semiconductor Magic-sized Clusters^8,35–37^ and the possibility aggregation of the sample during microscopy measurement, these as-synthesized PMSCs are in PMSCs range size with slightly smaller than other reports. Furthermore, the chemical composition of the PMSCs samples were characterized by Energy Dispersive X-ray Spectroscopy (EDS). Figure 3f showed the well-known elements of the C, O, Cs, Pb, and Br were identified in the CH_3_NH_3_PbBr_3_ PMSCs sample, implying the CH_3_NH_3_PbBr_3_ perovskite structure. While the Si element peak emerges for the CH_3_NH_3_PbBr_3_ PMSCs/SiO_2_ sample (Fig. 3g), suggesting incorporating of the SiO_2_ on the PMSCs sample. The corresponding elemental mapping of the selected area of CH_3_NH_3_PbBr_3_ PMSCs/SiO_2_ sample that marked in Fig. 3h, vividly shows the spatial distribution of the C, O, Cs, Pb, Br, and Si elements which are shown in (Fig. 3i–m).

The Effective Mass Approximation models proposed by Brus, was considered being applicable to PMSCs to estimate the bandgap energy of the PMSCs^9,38^ as follows

1 $$E_{{g\left( {PMSCs} \right)}} = E_{{g\left( {bulk} \right)}} + \frac{{h^{2} }}{{8r^{2} }} \left( {\frac{1}{{m_{e} }} + \frac{1}{{m_{h} }}} \right)$$

where $E_{{g\left( {PMSCs} \right)}}$ is the bandgap energy of the PMSCs, $E_{{g\left( {bulk} \right)}}$ is the bandgap energy of the bulk material, $h$ is the Planck’s constant, $r$ is the radius of the PMSCs, $m_{e}$ is the effective mass of the electron and $m_{h}$ is the effective mass of the hole. The bandgap energy of bulk properties was approximated by the Bulk CH_3_NH_3_PbBr_3_ ($E_{{g\left( {bulk} \right)}} = 2.30\;{\text{ eV}})$,^39,40^ and the effective mass of hole and electron was approximated by CH_3_NH_3_PbBr_3_ based PMSCs from a previous report ( $\left( {\frac{1}{{m_{e} }} + \frac{1}{{m_{h} }}} \right) = 2.37 \times 10^{30} \;{\text{ kg}}^{ - 1}$)^9^. Using this relation and the size of the samples from TEM measurement, the bandgap energy of the CH_3_NH_3_PbBr_3_ PMSCs and CH_3_NH_3_PbBr_3_ PMSCs/SiO_2_ are 2.49 and 2.39 eV, respectively. The precipitation process of PMSCs in the presence of SiO_2_ generate the CH_3_NH_3_PbBr_3_ PMSCs/SiO_2_ with the bandgap energy slightly smaller than the only CH_3_NH_3_PbBr_3_ PMSCs, owing to their quantum confinement effect. It was highly plausible because the hydroxyl groups on the SiO_2_ could attract the amine-based ligand on the precursors that have not been formed into PMSCs structure, surpassing the nucleation and growth process. Thus, the perovskite nanostructure's particle size with the SiO_2_ passivation is always bigger than the perovskite nanostructure itself that was also well-documented in other reports^19,41,42^.

### PL stability of CH_3_NH_3_PbBr_3_PMSCs and CH_3_NH_3_PbBr_3_ PMSCs/SiO_2_

**Figure 4:**
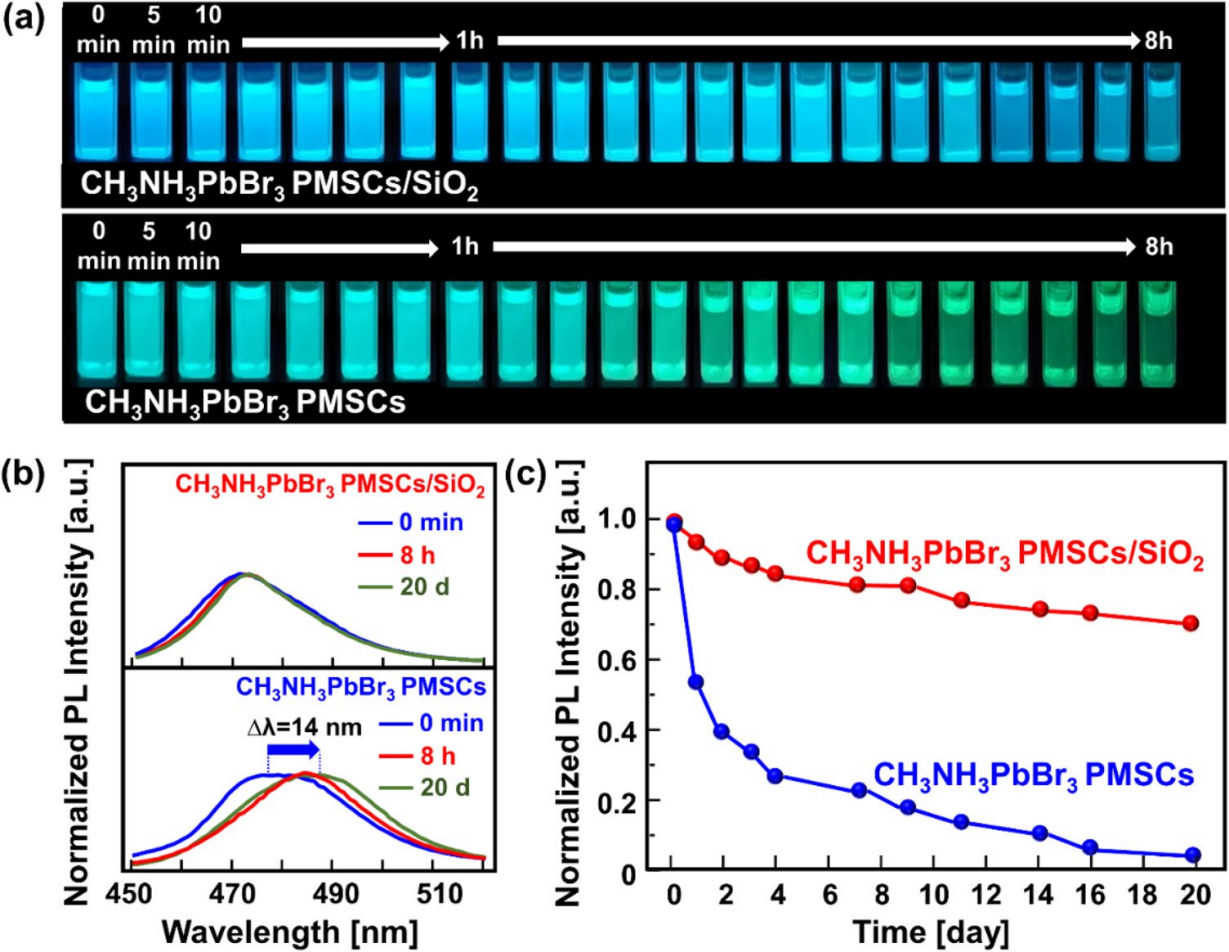
(**a**) Digital photograph of the CH_3_NH_3_PbBr_3_ PMSCs and the CH_3_NH_3_PbBr_3_ PMSCs/SiO_2_ under elapsed-time observation in ambient condition for 8 h. (**b**) The emission PL spectra of the representative samples. (**c**) PL intensity of the samples that observed in ambient condition for 20 days.

The environmental stability of the PMSCs is a major research interest. To evaluate their stability, the relative PL intensities of the CH_3_NH_3_PbBr_3_ PMSCs and CH_3_NH_3_PbBr_3_ PMSCs/SiO_2_ solutions were studied for 20 days in ambient conditions, with the results shown in Fig. 4. As shown in Fig. 4a, the PL emission of the CH_3_NH_3_PbBr_3_ PMSCs/SiO_2_ sample remains unchanged in blue emission for the first 8 h after synthesis process. While for the CH_3_NH_3_PbBr_3_ PMSCs sample, the PL emission is redshifted on the blue-green region. This PL quenching is probably attributed to the ligand detachments owing to protonation and deprotonation reaction, inducing aggregation of PMSCs^43,44^. Another possibility is that the growth process of the PMSCs is still ongoing because the spherical PMSCs have relatively higher surface energy and thus grow at a slower rate^45,46^.

Figure 4b shows the corresponding emission spectra of samples for 20 days of observation. The CH_3_NH_3_PbBr_3_ PMSCs/SiO_2_ sample emission peak remains constant, while the emission peak of the CH_3_NH_3_PbBr_3_ PMSCs was continuously redshifted as much of 14 nm. Figure 4c shows the PL intensity quenching with time for samples. The PL quenching of the CH_3_NH_3_PbBr_3_ PMSCs in air induced by chemical instability on the PMSCs surface^47^.

Even though the Oleic Acid and Oleylamine was used as ligand, the combination of both ligands promptly undergoes aggregation, sedimentation and degradation. This phenomenon is attributed to the high probability of proton transfer from oleic acid to Oleylamine, inducing the detachment of Oleylamine from the surface of the PMSCs^43,44^. Thus, the as-synthesized CH_3_NH_3_PbBr_3_ PMSCs suffered poor stability *i.e.*, degraded completely in 20 days indicated by the vanished luminescence in ambient condition. In contrast, the CH_3_NH_3_PbBr_3_ PMSCs/SiO_2_ exhibit a slow quenching in PL intensity and retained a high value (70% of the initial intensity) after 20 days. It is pointed out that the SiO_2_ enhanced the stability of the CH_3_NH_3_PbBr_3_ PMSCs, which should be due to the passivation of PMSCs surfaces.

**Figure 5:**
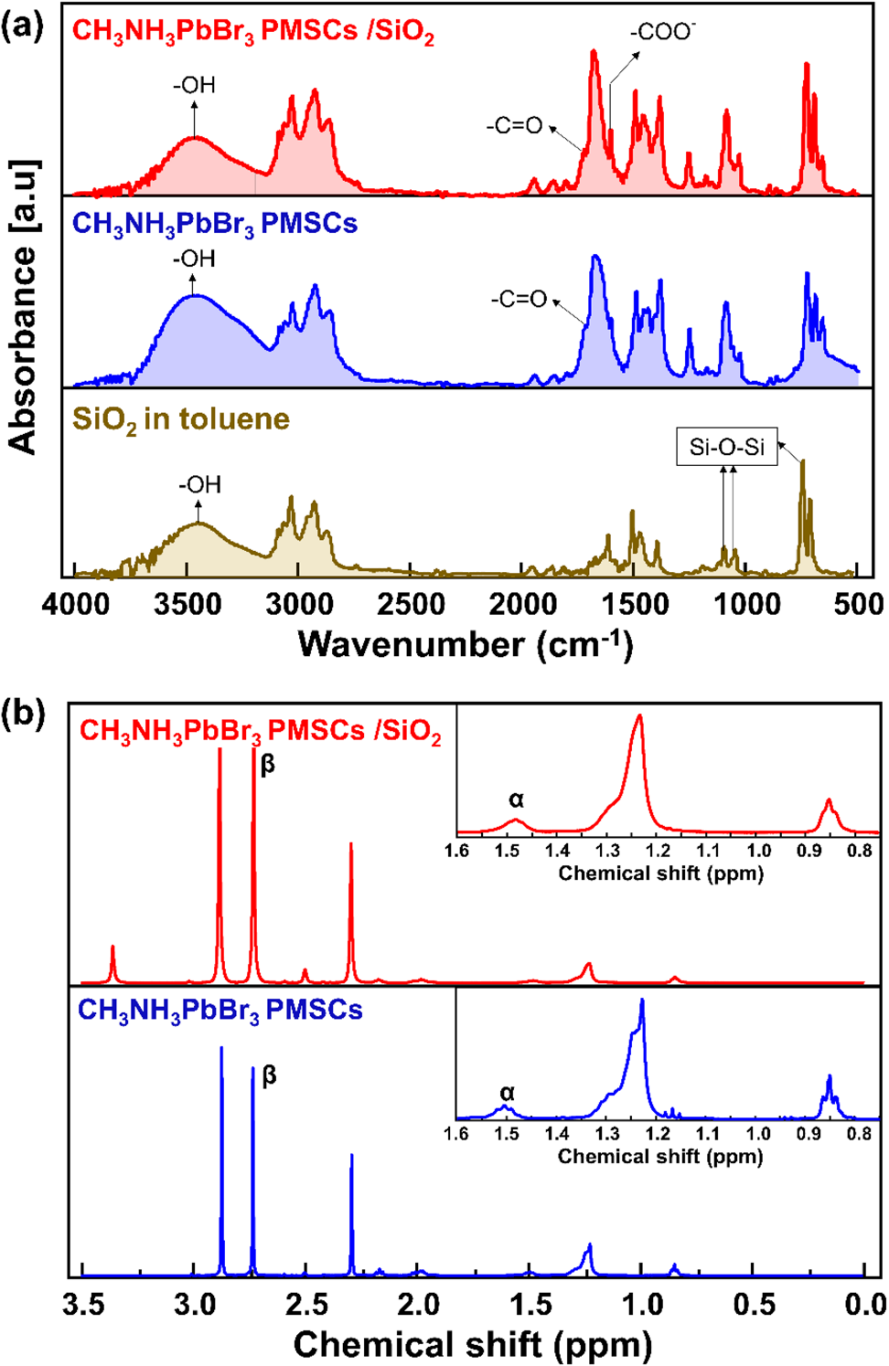
(**a**) FTIR Spectra of the SiO_2_ in toluene, CH_3_NH_3_PbBr_3_ PMSCs and CH_3_NH_3_PbBr_3_ PMSCs/SiO_2_. (**b**) ^1^H spectra of the CH_3_NH_3_PbBr_3_ PMSCs and CH_3_NH_3_PbBr_3_ PMSCs/SiO_2_ in deuterated DMSO. The inset figure is the magnification of the ^1^H spectra on the chemical shift range 0.8–1.6 ppm showing the broadening peaks for the CH_3_NH_3_PbBr_3_ PMSCs/SiO_2_.

The interaction mechanism of the PMSCs surface with the SiO_2_ was studied by Fourier Transform Infrared (FTIR). The FTIR spectra of the SiO_2_ dispersed in toluene, CH_3_NH_3_PbBr_3_ PMSCs and the CH_3_NH_3_PbBr_3_ PMSCs/SiO_2_ are shown in Fig. 5a. The FTIR spectrum of SiO_2_ in toluene demonstrates the strong peaks absorption at 800, 1087 and 1087 cm^−1^ that attributed to the Si–O–Si bonds. Also, the peak at 3430 cm^−1^ indicates the presence of Si–OH bonds on the SiO_2_ surfaces which is typically resulted interaction between toluene and the SiO_2_ surfaces^48,49^. In the CH_3_NH_3_PbBr_3_ PMSCs spectrum, the peak at 1729 cm^−1^ corresponds to C=O that a typically present in the Oleic Acid-modified Nanoparticle. The N–H stretching and C–C bond can be seen at around 3321 and 1652 cm^−1^, although the band overlaps with the –OH band, typically present in the Oleylamine-modified Nanoparticle^50^. It indicates that the as-synthesized CH_3_NH_3_PbBr_3_ PMSCs was well-capped by the Oleic acid and Oleylamine ligand. In addition of SiO_2_, the –COOH vibration peak at 1729 cm^−1^ and –OH vibration peak at 3430 cm^−1^ decreases, while a peak at 1590 cm^−1^ corresponding to COO^−^ (carboxylate) ions increase significantly. Thus, it could be suggested that the –C=O on the surface of CH_3_NH_3_PbBr_3_ PMSCs has reacted with the –OH on the surface of SiO_2_, resulted more carboxylate ions.

To investigate the interaction between the ligand species and the hydroxyl groups on the surface of SiO_2_, the ^1^H NMR solution was conducted. Comparing with the ^1^H spectra of the used ligand species (Fig. S5), Fig. 5b shows the characteristics resonances of the Oleic acid and Oleylamine in all samples, suggesting a well-capping of the PMSCs by these ligands consistent with the FTIR spectra in Fig. 5a. It is worth to noting that the α resonances has been shifted from 1.474 ppm from the CH_3_NH_3_PbBr_3_ PMSCs to 1.490 ppm for CH_3_NH_3_PbBr_3_ PMSCs/SiO_2_, indicating the amine-groups on the perovskite surface interact with the silica surfaces^51^. Furthermore, we have evaluated the ^1^H spin–spin (T_2_) relaxation measurement that is sensitive to the dynamically dipole–dipole interaction between molecules in solvent^46,52,53^. The T_2_ relaxation time attributes to the required time of the molecules in the solution to return to an initial equilibrium state after a dynamic motion by an electromagnetic radiation^52^. The spin–spin (T_2_) relaxation measurement (Table S1) shows that all ^1^H NMR signal in the range of 0–3.5 ppm of the CH_3_NH_3_PbBr_3_ PMSCs/SiO_2_ has faster relaxation times than those CH_3_NH_3_PbBr_3_ PMSCs. The detailed implication of the T_2_ relaxation time will be discussed further in discussion section.

## Discussion

PMSCs structure typically suffers a metastable phase since their poor passivation due to their smaller size and large surface/volume ratio. In a common pathway, the PMSCs formation during precipitation is reversible with the PQDs formation^8,30^. While the PQDs easily undergo aggregation, sedimentation, and degradation in ambient condition. A poor stability of PMSCs corresponds to its surface defect on Pb^2+^, Br^−^, and CH_3_NH_3_^+^ sites. In CH_3_NH_3_PbBr_3_ PMSCs sample, a high concentration of the oleic acid and oleylamine are used in conjunction ligands. The amine-based ligand was usually used to passivate the Pb^2+^ and CH_3_NH_3_^+^ defects by exploits a lone pair electron of the N atoms. An excessive amine-based ligand could generate more ammonium cation $\left( {NH_{3}^{ + } } \right)$, to passivate the Br^−^ defects on the surfaces. In addition, amine-based ligand can deprotonate carboxylic acid (from Oleic Acid) into carboxylate ion, $COO^{ - }$ that should effectively passivate Pb^2+^ and CH_3_NH_3_^+^ defects. The protonation-deprotonation reaction between Oleic acid and Oleylamine ligand that might be possibly occurred can be described by Eq. (2)

2 $$R - COOH + R^{\prime } - NH_{2} \leftrightarrow R - COO^{ - } + R^{\prime } - NH_{3}^{ + }$$

**Figure 6:**
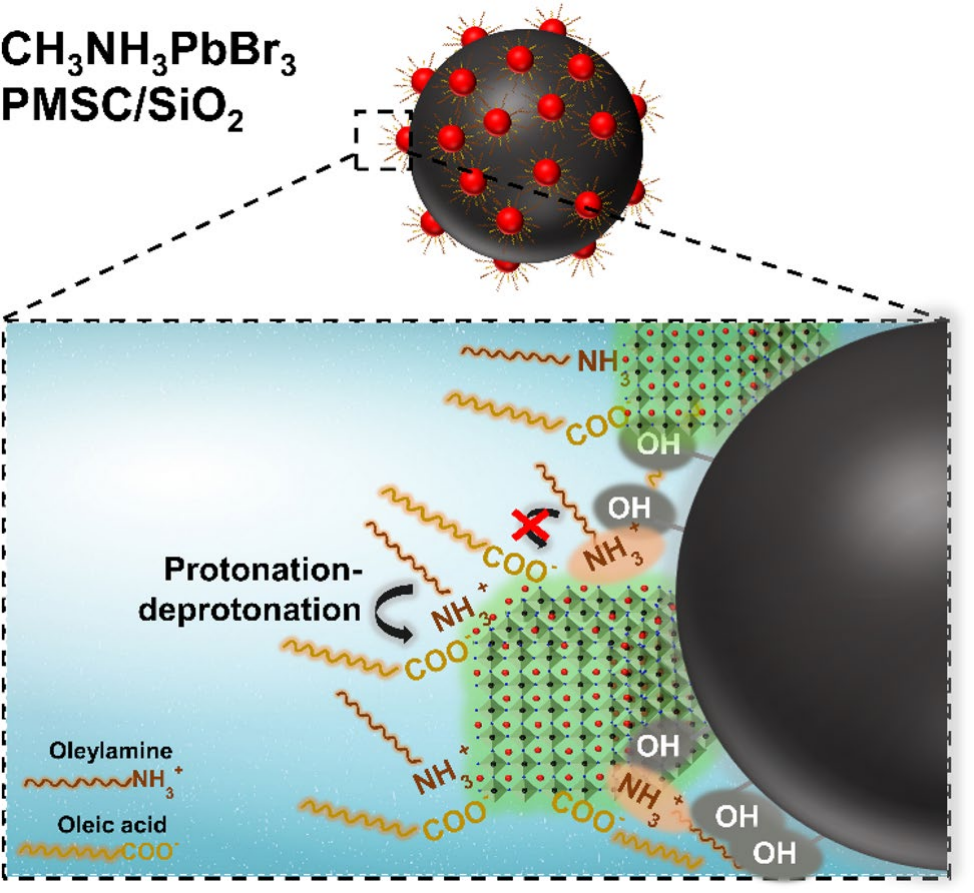
Illustration of the surface passivation of the CH_3_NH_3_PbBr_3_ PMSCs by SiO_2_ and oleylamine and oleic acid ligands.

However, a reversible protonation-deprotonation reaction could increase the possibility of the ligand detachment from the PMSCs surface, leading a poor stability. Addition of the SiO_2_ enriched with OH groups on the surfaces to the system, induced the chemical reaction with the $R - COOH$ and R-NH_2_ via dipole–dipole interaction as observed by the chemical shift of the ^1^H spectra of the α and β resonance to the higher values that was indicated by the chemical shift toward higher value (Fig. 5b)^51,54^. This interaction shifted the equilibrium protonation-deprotonation reaction into other side, generating more carboxylate ions, $COO^{ - }$ ^49^. An abundant carboxylate ion which was detected by the increased absorbance IR spectrum at 1590 cm^−1^ as shown in Fig. 5a, is expected to effectively passivate PMSCs surface defect. The spherical PMSCs has relatively higher surface energy and thus grows at a slower rate^45^. Then, the addition of the SiO_2_ that has a high specific surface area, inducing the growth PMSCs that is still undergoing are prone to attach to the surface of SiO_2_^18^. The PMSCs attached on the SiO_2_ surface via both ion–dipole and dipole–dipole interaction, was also observed as the faster (lower T_2_ value) of the overall T_2_ relaxation times of the CH_3_NH_3_PbBr_3_ PMSC/SiO_2_ in the range of 0–3.5 ppm (Table S1). The faster of the T_2_ relaxation times, the more PMSC are non-covalently bound on the SiO_2_ surface^53^. Thus, the PMSCs attachment on the surfaces helps ensure the crystal structure in humid condition and prevent the PL quenching of these PMSCs. The surface passivation of the PMSCs by SiO_2_ and an excessed surface ligand was illustrated in Fig. 6.

## Conclusions

In summary, CH_3_NH_3_PbBr_3_ PMSCs was synthesized by utilising of an excess concentration of capping ligands i.e., oleylamine and oleic acid and SiO_2_ addition through a ligand assisted re-precipitation method. The synergetic effect of the ligand and SiO_2_ was investigated systematically. For the PMSCs samples without SiO_2_ addition, the PL emission was vanished, while with the SiO_2_ addition, the PL emission retained 70% of its initial emission intensity in ambient condition for 20 days. FTIR analysis was conducted to investigate the surface ligand composition and the underlying mechanism. We suggest that the SiO_2_ enriched with OH groups on the PMSCs surfaces induced the chemical reaction with the $R - COOH$ generating more carboxylate ion, $COO^{ - }$. These abundant carboxylate ions could effectively passivate the PMSCs surface by inhibiting the protonation-deprotonation between the amine and acid-based ligands, lead an enhanced PL stability than the PMSCs without SiO_2_. This study provides a deeper insight into the metastability phenomenon of the PMSCs at ambient condition. It has important implications in understanding and controlling nucleation and growth of semiconductor nanoparticles through a reliable characterization of PMSCs.

## Methods

### Materials

Lead(II) Bromide (PbBr_2_, ≥ 98%, Sigma Aldrich, Singapore Ltd.), Methylamine Bromide (CH_3_NH_3_PbBr_3_, 98%, Sigma Aldrich, Singapore Ltd.), Oleylamine (C_18_H_35_NH_2_, 70%, Sigma Aldrich, Singapore), Oleic acid (C_18_H_3_O_2_, Sigma Aldrich, Singapore), Anhydrous N,N-Dymethylformamide (C_3_H_8_N_2_, Merck Ltd., Indonesia), Toluene (C_7_H_8_, Merck Ltd., Indonesia Ltd.), Fumed Silica (SiO_2_, 98%, Aerosil 380, Evonik Ltd., Singapore). All materials were used without further purification.

### Synthesis of CH_3_NH_3_PbBr_3_ PMSCs

CH_3_NH_3_PbBr_3_ PMSCs was synthesized by a ligand assisted co-precipitation that was reported elsewhere^26^. In brief, a mixture containing of 0.4 mmol of PbBr2 that was dissolved in dimethylformamide (DMF), 0.4 mmol oleylamine, 3 mmol oleic acid, and 0.32 mmol CH_3_NH_3_Br_2_ in DMF was sonicated by an ultra-sonication. Then, 10% (v/v) of the precursor was precipitated in toluene that was precooled or preheated under vigorous stirring. Precipitation temperature was varying to obtain the tunable emission of PMSCs. The obtained solution turned into thick yellow color and exhibited a luminescence. Centrifugation was conducted to remove the by-product from the obtained the colloidal CH_3_NH_3_PbBr_3_ PMSCs. A syringe filtration 0.22 µm (RC membrane, Satorius Co.) was performed as the last step of sample purification for further characterizations.

### Synthesis of CH_3_NH_3_PbBr_3_ PMSCs /SiO_2_

A fumed silica, SiO_2_ (10 wt.%) was dispersed in toluene under vigorous stirring at room temperature. Subsequently, 20% (v/v) of SiO_2_ mixture was added into the precipitated PMSCs immediately, resulted CH_3_NH_3_PbBr_3_ PMSCs/SiO_2_. Centrifugation was conducted to remove the by-product from the obtained the colloidal CH_3_NH_3_PbBr_3_ PMSCs/SiO_2_. A syringe filtration 0.22 µm (RC membrane, Satorius Co.) was performed as the last step of sample purification for further characterizations.

### Characterization

Ultraviolet–visible (UV–Vis) absorbance spectra were measured by an Ocean Optic, D-2000 using a quartz cuvette with 10 mm optical path length at room temperature. The PL spectra of the samples were measured by an Agilent Cary eclipse spectrofluorometer with a Xenon lamp as light source. Transmission Electron Microscopy (TEM) characterization was conducted by drop-casting samples solution on a commercial EM Grid with Copper coating operated at acceleration voltage 120 kV using Hitachi HT7700. Energy-dispersive X-ray spectroscopy (EDS) was conducted by JEOL JSM 6510 operated at acceleration voltage 15 kV. The sample solution was dropped onto a KBr pellet to conduct the Fourier Transform Infra-Red (FTIR) measurement using IR Prestige-21 FTIR Spectrometer (Shimadzu, a spectra resolution of 1 cm^−1^). ^1^H NMR spectra was recorded by 500 MHz NMR Agilent DD2 Spectrometer (Agilent Technologies) in deuterated DMSO.

## Supplementary Information

Supplementary Information.
