# Supplementary material for: Synergetic effect of the surface ligand and SiO2 driven photoluminescence stabilization of the CH3NH3PbBr3 perovskite magic-sized clusters
Source: Sci Rep. 2021 Nov 15;11:22211. doi: 10.1038/s41598-021-01560-4 (PMC8593060; doi:10.1038/s41598-021-01560-4)
Supplement: Supplementary file 1 — Supplementary Information. [file 41598_2021_1560_MOESM1_ESM.pdf]

**Electronic supplementary material**

# Synergetic Effect of the Surface Ligand and $SiO_2$ Driven Photoluminescence Stabilization of the $CH_3NH_3PbBr_3$ Perovskite Magic-Sized Clusters

*Fitri Aulia Permatasari*[†], *Hilma Eka Masitoh*[†], *Ea Cahya Septia Mahen*[†,‡], *Bebeh Wahid Nuryadin*[§], *Akfiny Hasdi Aimon*[†], *Yana Maolana Syah*[ξ] *and Ferry Iskandar*[†,ϕ,*]

*E-mail: ferry@fi.itb.ac.id

[†]Department of Physics, Faculty of Mathematics and Natural Sciences, Institut Teknologi Bandung, Jalan Ganesha 10, Bandung, West Java, Indonesia 40132

[‡]Department of Physics Education, Faculty of Tarbiyah and Education, UIN Sunan Gunung Djati Bandung, Jl. A. H. Nasution 105 Bandung Indonesia 40614

[§]Department of Physics, Faculty of Science and Technology, UIN Sunan Gunung Djati Bandung, Jl. A. H. Nasution 105 Bandung Indonesia 40614

[ξ] Department of Chemistry, Faculty of Mathematics and Natural Sciences, Institut Teknologi Bandung, Jalan Ganesha 10 Bandung, Indonesia 40132

[ϕ]Research Center for Nanosciences and Nanotechnology, Institut Teknologi Bandung, Jalan Ganesha 10 Bandung, Indonesia 40132

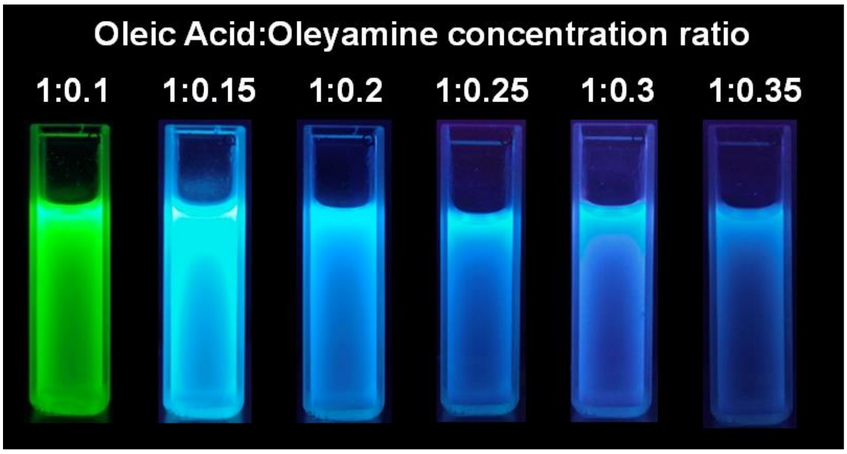


**Figure S1**. Digital image of the perovskite samples that was synthesized in varying ligands concentration ratio at room precipitation temperature.

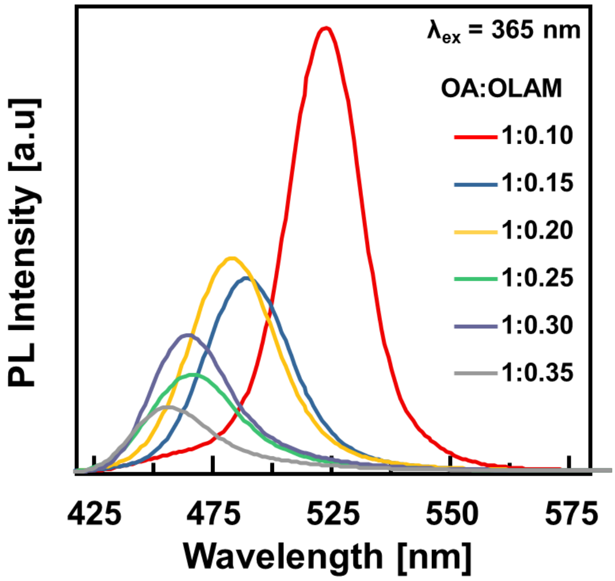


**Figure S2**. PL emission spectra of the perovskite samples that was synthesized in varying ligands concentration ratio at room precipitation temperature.

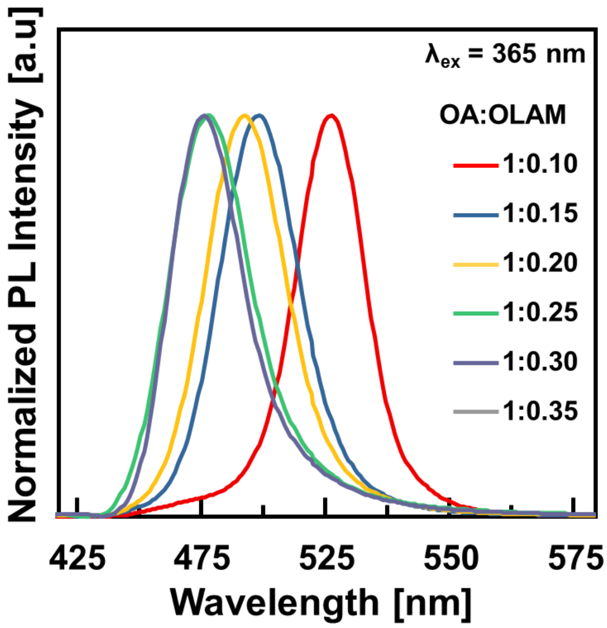


**Figure S3**. Normalized PL emission spectra of the perovskite samples that was synthesized in varying ligands concentration ratio at room precipitation temperature.

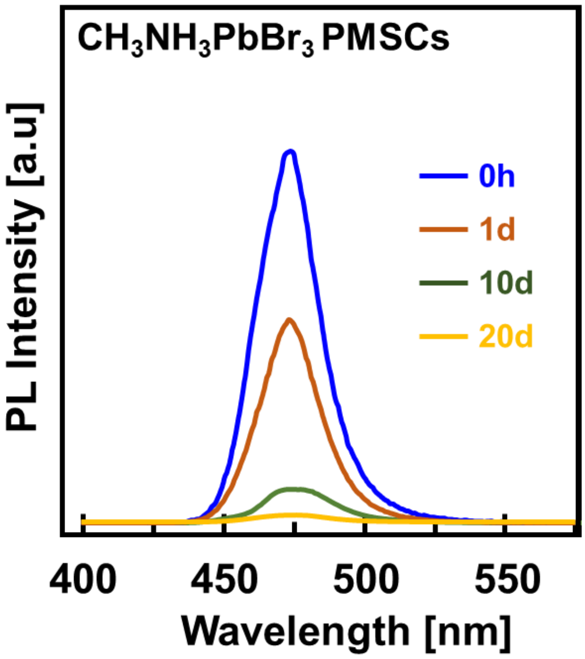


**Figure S4**. Photoluminescence spectra of the $CH_3NH_3PBBr_3$ PMSC that was stored in ambient condition for 20 days.

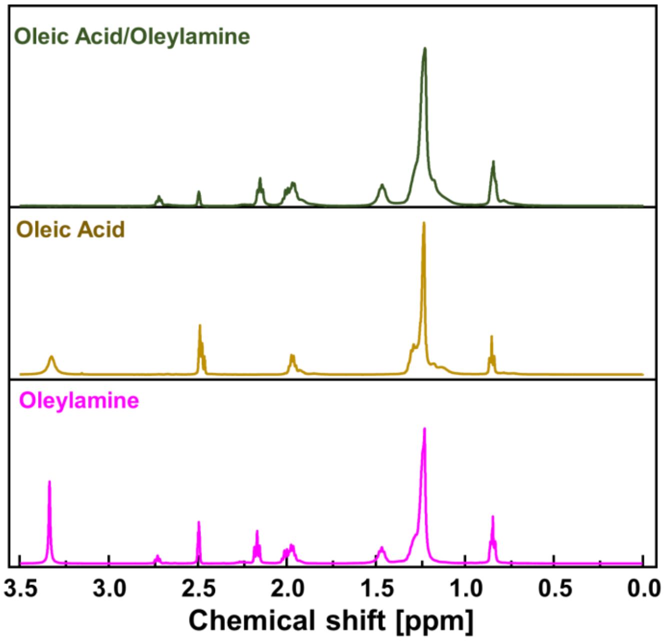


**Figure S5**. $^{1}$H NMR spectra of the ligands that used.

**Table S1**. $T_2$ relaxation times of the PMSCs samples that corresponds to the 1H NMR spectra in Figure 5b.

| $CH_3NH_3PbBr_3$ PMSC | | $CH_3NH_3PbBr_3$ PMSC/$SiO_2$ | |
|---|---|---|---|
| **Chemical shift (ppm)** | **$T_2$ relaxation time (s)** | **Chemical shift (ppm)** | **$T_2$ relaxation time (s)** |
| 0.844 | 1.623 | 0.844 | 0.167 |
| 1.222 | 4.587 | 1.225 | 4.058 |
| 1.474 | 0.135 | 1.490 | 0.061 |
| 1.981 | 0.149 | 1.978 | 0.081 |
| 2.286 | 5.182 | 2.289 | 3.878 |
| 2.724 | 15.070 | 2.727 | 8.771 |
| 2.866 | 3.629 | 2.876 | 3.472 |
| 3.001 | 4.076 | 3.011 | 3.629 |
| 3.565 | 0.580 | 3.352 | 0.257 |
